# Ursodeoxycholic acid in the management of symptomatic gallstone disease: systematic review and clinician survey

**DOI:** 10.1093/bjsopen/zrac152

**Published:** 2023-03-23

**Authors:** Lewis Hall, James Halle-Smith, Richard Evans, Giles Toogood, Tom Wiggins, Sheraz R Markar, Spyros Kapoulas, Paul Super, Olga Tucker, Siobhan C McKay

**Affiliations:** College of Medical and Dental Scientists, University of Birmingham, Birmingham, UK; Liver Unit, Queen Elizabeth Hospital Birmingham, University Hospitals Birmingham UK, Birmingham, UK; Department of Academic Surgery, University of Birmingham, Birmingham, UK; Department of Hepatobiliary Surgery, St James’ Hospital, Leeds, UK; Department of UGI Surgery, Heartlands Hospital, University Hospitals Birmingham, Birmingham, UK; Nuffield Department of Surgery, University of Oxford, Oxford, UK; Department of UGI Surgery, Heartlands Hospital, University Hospitals Birmingham, Birmingham, UK; Department of UGI Surgery, Heartlands Hospital, University Hospitals Birmingham, Birmingham, UK; Department of UGI Surgery, Heartlands Hospital, University Hospitals Birmingham, Birmingham, UK; Liver Unit, Queen Elizabeth Hospital Birmingham, University Hospitals Birmingham UK, Birmingham, UK; Department of Academic Surgery, University of Birmingham, Birmingham, UK

## Abstract

**Background:**

Symptomatic gallstones are common. Ursodeoxycholic acid (UDCA) is a bile acid that dissolves gallstones. There is increasing interest in UDCA for symptomatic gallstones, particularly in those unfit for surgery.

**Method:**

A UK clinician survey of use and opinions about UDCA in symptomatic gallstones was performed, assessing clinicians’ beliefs and perceptions of UDCA effectiveness. A systematic review was performed in accordance with the PRISMA guidelines. PubMed, MEDLINE, and Embase databases were searched for studies of UDCA for symptomatic gallstones (key terms included ‘ursodeoxycholic acid’; ‘UDCA’; ‘biliary pain’; and ‘biliary colic’). Information was assessed by two authors, including bias assessment, with independent review of conflicts.

**Results:**

Overall, 102 clinicians completed the survey, and 42 per cent had previous experience of using UDCA. Survey responses demonstrated clinical equipoise surrounding the benefit of UDCA for the management of symptomatic gallstones, with no clear consensus for benefit or non-benefit; however, 95 per cent would start using UDCA if there was a randomized clinical trial (RCT) demonstrating a benefit. Eight studies were included in the review: four RCTs, three prospective studies, and one retrospective study. Seven of eight studies were favourable of UDCA for biliary pain. Outcomes and follow-up times were heterogenous, as well as comparator type, with only four of eight studies comparing with placebo.

**Conclusion:**

Evidence for UDCA in symptomatic gallstones is scarce and heterogenous. Clinicians currently managing symptomatic gallstone disease are largely unaware of the benefit of UDCA, and there is clinical equipoise surrounding the benefit of UDCA. Level 1 evidence is required by clinicians to support UDCA use in the future.

## Teaser

Ursodeoxycholic acid (UDCA) may be beneficial for patients with symptomatic gallstone disease; however, current evidence is heterogenous. Clinicians currently managing symptomatic gallstone disease are largely unaware of the benefit of UDCA, and there is clinical equipoise surrounding the benefit of UDCA. Level 1 evidence is required by clinicians to support UDCA use in the future.

## Introduction

Gallstones are common, and their incidence increases with age to 30–50 per cent of those older than 70 years^1^. One-third of patients with gallstones develop symptoms, ranging from biliary colic to cholecystitis, cholangitis, and pancreatitis. The current standard management for symptomatic gallstone disease is a laparoscopic cholecystectomy, one of the most common abdominal operations performed in the UK, with more than 66 000 performed annually^2,3^.

Older patients are often deemed unfit for cholecystectomy due to multimorbidity, frailty, and reduced physiological reserve and undergo non-operative management (NOM). NOM consists of dietary advice, analgesia, antibiotics, and biliary drainage if required^4^; however, NOM is associated with persistent and recurrent symptoms, multiple hospital admissions, and death from sepsis^5^. A significantly worse clinical outcome is seen in older patients after laparoscopic cholecystectomy with increasing age^6^. There is an urgent need to consider effective, alternative non-surgical approaches to management. Surgical waitlists are at their longest ever in the UK following the COVID-19 pandemic, with more than 5 million patients waiting for surgery, and this patient cohort would benefit from optimization of NOM^7^.

Ursodeoxycholic acid (UDCA) is an oral bile acid that reduces cholesterol secretion by lowering the cholesterol concentration of bile in the gallbladder. It dissolves gallstones and is licenced for use^8^; however, due to the widespread adoption of laparoscopic cholecystectomy and lack of clinician awareness of its utility, the current use of UDCA for symptomatic gallstones is uncertain.

The hypothesis of the present study was that UDCA could be beneficial in reducing symptoms in patients with gallstone disease, improving NOM of symptomatic gallstones in patients deemed too high risk for surgery, such as the elderly and/or those with significant co-morbidities. The aim was to assess current UK clinician knowledge, use, and opinion of UDCA in managing symptomatic gallstone disease. In addition, a systematic review of the published literature on the use of UDCA in symptomatic gallstone disease was performed to ascertain the current level of evidence pertaining to UDCA effect in reducing biliary pain in patients with symptomatic gallstone disease.

## Methods

### Clinician survey

This study is a nationwide electronic survey that was carried out between May and July 2021. The survey was constructed on a web-based platform (Google Surveys^®^) and distributed via e-mail to members of the Association of Upper Gastrointestinal Surgery (AUGIS), and on the Twitter social media platform using hashtags #gallstones and #frailty. It was designed to target consultant and trainee clinicians working within the National Health Service (NHS) across the UK. AUGIS members received an official e-mail invitation with the survey details, and a reminder e-mail was sent 1 month later. Participants were informed in advance that the survey would ask questions about UDCA use in symptomatic gallstones, whether used or not, to establish current UDCA use nationally, and aimed to establish whether there is sufficient equipoise for a future trial. To ensure participants were clinicians treating patients with symptomatic gallstones several demographic questions were asked (for example name, e-mail address, hospital of clinical practice, clinical grade, and specialty), and details regarding their and their hospitals clinical experience surrounding management of symptomatic gallstones. No rewards were offered for participation in the survey.

### Survey questions

The survey was designed to assess clinical practice in the NOM of symptomatic gallstones, assessment of fitness for surgery, UDCA use (dosing, duration, and follow-up), and beliefs and perceptions in relation to UDCA effectiveness and clinically important outcomes to support UDCA use. The rationale was to explore in detail, the differences in individual practices regarding the assessment of patients unfit for surgery, assess current UDCA practice, and establish equipoise and clinically meaningful outcomes for a trial of UDCA for NOM of symptomatic gallstones. Therefore, the survey questionnaire was divided into two sections: regarding NOM of patients with symptomatic gallstones, and then UDCA use and perceptions. The full survey is available in the *Supplementary material*.

### Statistical analysis

Frequencies and percentages were used to summarize survey responses. Missing data were captured and examined.

## Systematic review

### Design

The review was designed and performed in accordance with PRISMA guidelines^9^. The PubMed, MEDLINE, and Embase databases were searched in July 2021 for published studies reporting the use of UDCA for symptomatic gallstones. Inclusion criteria for studies included adult patients with symptomatic gallstones, and the use of UDCA for treatment. The outcome measure was biliary pain. No date restrictions were used in the search. Reference lists of relevant studies were also cross-referenced to identify additional studies. Key terms related to UDCA, gallstones, and their sequalae were used to complete the search (including, but not limited to ‘ursodeoxycholic acid’, ‘UDCA’, ‘biliary pain’, and ‘biliary colic’). The complete search terms are available in the *Supplementary material*. Case reports, literature reviews, animal studies, studies that investigated the paediatric or obstetric populations, and studies that did not provide a quantitative assessment of impact of UDCA on symptoms of gallstones were excluded. Data were included from randomized clinical trials (RCTs) if UDCA was compared with standard care or placebo. Where previous standard care was no longer acceptable (for example chenodeoxycholic acid (CDCA) use), single-arm data were assessed if pre- and post-UDCA results were presented. Studies investigating the use of UDCA in patients undergoing bariatric surgery who did not have gallstones were excluded. Two reviewers (L.H. and J.H.S.) independently identified the studies for inclusion. Any discrepancies were identified and resolved through discussion and third-party involvement (S.M.).

### Data collection

The primary endpoints were to assess the impact of UDCA on biliary pain in patients with gallstones and to determine current consensus on the use of UDCA for symptomatic gallstone disease. Study characteristics collected were country of publication, publication year, and sample size. Evidence grade of recommendations, assessment, development, and evaluation (GRADE) was assessed according to the Cochrane GRADE approach^10^.

### Bias assessment

Assessment of risk of bias was completed by two authors (L.H. and J.H.S.). The quality of RCTs was assessed in accordance with guidance from the Cochrane collaboration (*Fig. 4*), and the quality of cohort studies was assessed using the Newcastle–Ottawa scale (NOS) (*Fig. 5*)^9,11^.

## Results

### Clinician survey

#### Characteristics of survey respondents

All 102 respondents were involved in the care of patients with symptomatic gallstones in the UK. Respondents had a spectrum of clinical experience, from higher level trainees (ST3–ST8/senior fellow, 26 per cent), to consultants (74 per cent; 1–2 years as a consultant (*n* = 12); 3–5 years (*n* = 13); 5–10 years (*n* = 16); and more than 10 years (*n* = 34)).

#### Previous experience of UDCA for symptomatic gallstone disease

**Fig. 1:**
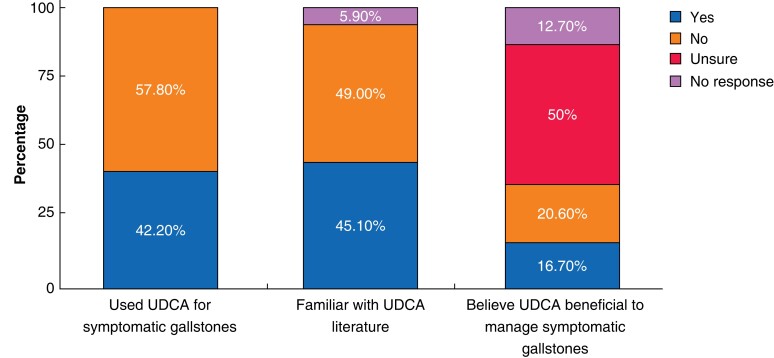
Survey respondents previous experience of UDCA use for treatment of symptomatic gallstones, knowledge of literature for UDCA effectiveness in symptomatic gallstones, and clinician belief of benefit for management of symptomatic gallstones in those non-operatively managed due to high surgical risk UDCA, ursodeoxycholic acid.

Experience of UDCA use varied among centres and clinicians, with 42 per cent having previous experience of UDCA in the management of symptomatic gallstone disease (*Fig. 1*). All clinicians using UDCA used it for definitive NOM (lifelong or until gallstone dissolution), and 12 of 43 (28 per cent) used UDCA in patients with symptomatic gallstones while undergoing optimization for surgery (for example weight management) (*Table 1*). Follow-up of patients after UDCA commencement was variable, with 28 of 42 (67 per cent) reporting routine follow-up to assess efficacy or side effects.

**Table 1:** Survey respondents previous experience of ursodeoxycholic acid use for symptomatic gallstones

Previous UDCA clinical experience		*n* (%)
**Have you used UDCA in treatment of symptomatic gallstones?**		
(*n* = 102)	Yes	43 (42)
	No	59 (58)
Aim of UDCA treatment* (could select multiple options)		
(*n* = 43)	Definitive non-operative treatment: lifelong prescription	29 (67)
	Definitive non-operative treatment: time-limited prescription (short-term or until stone dissolution)	20 (47)
	Symptomatic treatment while optimizing for surgery (for example for weight management)	12 (28)
**Do you follow-up patients you have started on UDCA to assess efficacy and side effects?**		
(*n* = 42)	Yes	28 (67)
	No	14 (33)
	Missing	1
**Type of follow-up after commencing UDCA?**		
(*n* = 27)	Clinical assessment	26 (96)
	Imaging	6 (22)
	(both clinical assessment and imaging)	(5)
	Missing	1

UDCA, ursodeoxycholic acid.

#### Clinician perspectives of effect of UDCA and future use

**Fig. 2:**
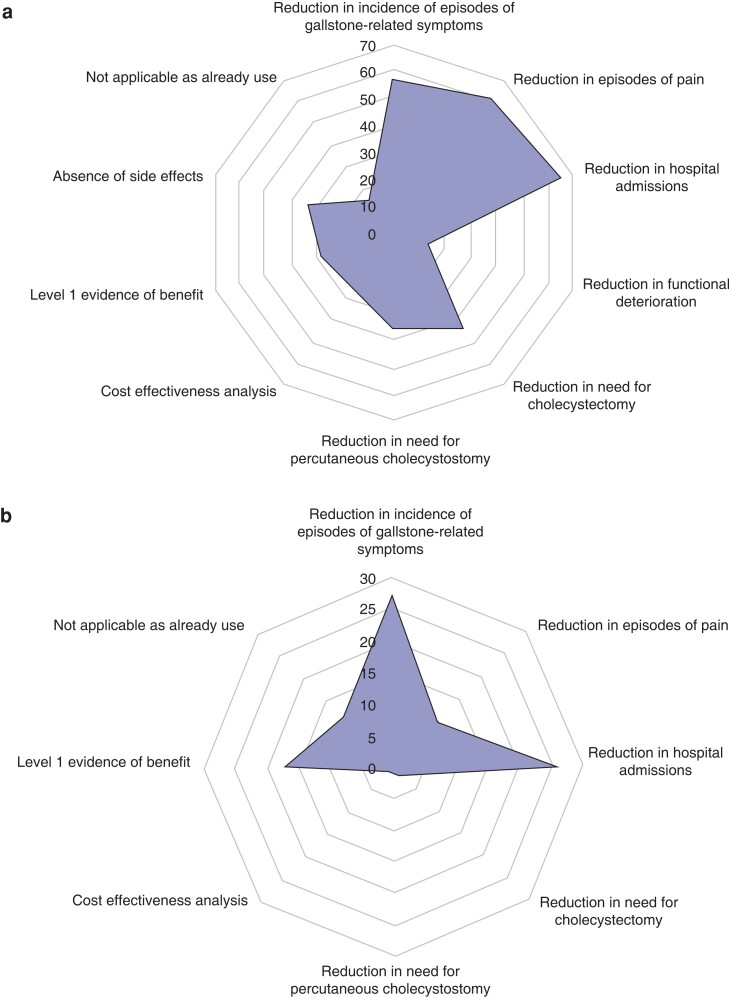
Survey responses from clinicians as what evidence would be required by to adopt UDCA into routine practice for the non-operative management of patient with symptomatic gallstones deemed high risk for surgery **a** What evidence would be required? **b** Most important piece of evidence required. UDCA, ursodeoxycholic acid.

Survey responses demonstrated clinical equipoise surrounding the benefit of UDCA for the management of symptomatic gallstones, with no clear consensus for benefit or non-benefit. Nineteen per cent believed that UDCA could be beneficial, whereas more than half were unsure of the benefit of UDCA (57 per cent) (*Table 2*). When asked about specific benefits, 59 responses were received, of which 63 per cent perceived benefits, including reduction in gallstone size, reduction in episodes of biliary pain, and reduction in hospital admissions. Overall, 54 responders (53 per cent) indicated their views on the reasons for not using UDCA in patients with symptomatic gallstone disease (*Table 2*), the most frequent being a belief that it simply did not work; however, almost all said that they would start using UDCA if there was an RCT demonstrating benefit, and two-thirds responded that the most clinically relevant outcome in such a trial would be a reduction in the number of hospital admissions with gallstone-related symptoms, followed by a reduction in the incidence of gallstone-related symptoms (*Fig. 2*).

**Table 2:** Survey respondents beliefs and knowledge regarding ursodeoxycholic acid use for symptomatic gallstones, with reference to patients managed non-operative as deemed too high risk for surgery

Beliefs and knowledge about UDCA		*n* (%)
**Do you believe that UDCA can be beneficial in the management of symptomatic gallstones?**		
(*n* = 89)	Yes	17 (19)
	No	21 (24)
	Unsure	51 (58)
	Missing	13
**What do you think are the beneficial effects of UDCA in symptomatic gallstone disease? (could select multiple options)**		
(*n* = 59)	N/A (do not believe it works)	22 (37)
	Reduction in hospital admissions	18 (31)
	Reduction in pain	16 (27)
	Reduction in size of gallstone	26 (44)
	Missing	43
**If you do not use UDCA in symptomatic gallstone disease, why do you not use it? (could select multiple options)**		
(*n* = 54)	Do not believe it works	38 (70)
	Unaware of use in gallstone disease	12 (22)
	Unacceptable side-effect profile	10 (19)
	Concerns about polypharmacy	1 (2)
	Manage all patients operatively	5 (9)
	Too expensive	0 (0)
	Missing	10
**Are you familiar of the literature concerning the use of UDCA and gallstones?**		
(*n* = 96)	Yes	46 (48)
	No	50 (52)
	Missing	6
**Would you start using UDCA if there was an RCT demonstrating its benefits?**		
(*n* = 101)	Yes	97 (96)
	No	4 (4)

UDCA, ursodeoxycholic acid; RCT, randomized clinical trial.

## Systematic review

**Fig. 3:**
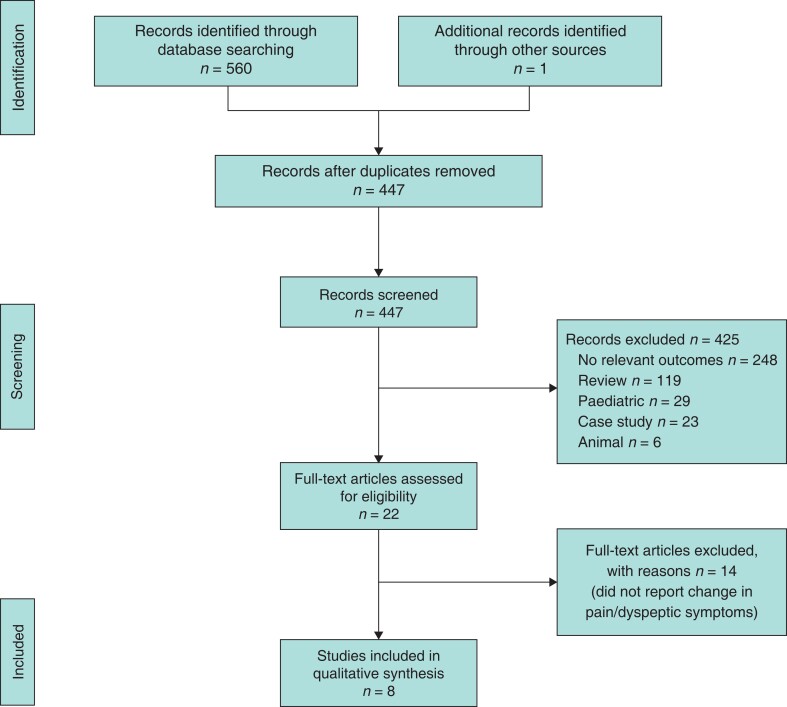
PRISMA flow chart

The literature search yielded 560 records, of which 446 titles and abstracts were screened after duplicates were removed. Full-text screening was performed for 21 records. One further study was identified from manual screening of the reference lists of included studies. Eight studies were included in the final systematic review: four RCTs, three prospective cohort studies, and one retrospective review (*Fig. 3*).

*Table 3* summarizes the key findings from each study, highlighting the GRADE score and reduction in episodes of biliary pain. Seven of the eight studies suggest that UDCA may be beneficial in reducing biliary pain in patients with gallstones. Of these studies, one (Petroni *et al.*) received a ‘high’ GRADE score, one received a ‘moderate’ score, and the remaining five had a ‘low’ score. One study was unfavourable for UDCA use (Venneman *et al.*) and had a moderate GRADE score.

**Table 3:** Summary table of studies included in systematic review

Patient: adults with symptomatic gallstone disease; Setting: hospital, outpatients; Intervention: UDCA treatment; Comparison: usual care or placebo where possible; Outcome: biliary pain/colic episodes									
Author (Country)	Design	(1) No. of patients	Age (years), mean(s.d.)	Sex ratio (M:F)	(1) Follow-up	Outcome and results	Opinion on UDCA	Quality of the evidence (GRADE)	Comments
		(2) Gallstone cohort			(2) UDCA dose				
		(3) Intervention							
		(4) Comparator							
Venneman (Netherlands)^12^	RCT	177 (SG)			77 days (7–244)	Colic episodes in 6 months	Unfavourable	Moderate	Short treatment duration
		(I) UDCA (89 of 177)	47(1)	1:4	750mg/d	11 of 81 (14%)			
		(C) Placebo (88 of 177)	45(1)	3:7		6 of 81 (7%)			
Polli (Italy)^13^	RCT	42 (SG)	Adults	1:3.2	14 days	Dyspeptic symptoms at 0, 7, and 14 days	Favourable	Low	Very short treatment duration
		(I) UDCA (23 of 42)			300mg/d	(1) Severity (19 patients): mean score reduced 6.00 to 2.11 to 0.84			
						(2) Episodes (23 patients): average score reduced 5.61 to 2.57 to 1.39			
		(C) Placebo (19 of 42)				(1) Severity (19 patients) 5.84 to 4.05 to 3.47			
						(2) Episodes (16 patients) 5.75 to 4.13 to 3.06			
Frigerio (Italy)^14^	RCT	240 (SG)	Adults		14 days	(1) Reduction dyspeptic symptoms (2) Reduction pain symptoms	Favourable	Moderate	Very short treatment duration
									
		(I) UDCA		7:13	300 mg/d	(1) of 120 (83%) (2) 82 of 101 (81%)			
		(C) Placebo		19:41		(1) 65 of 120 (54%) (2) 47 of 99 (47%)			
Petroni (UK/Italy)^15^	RCT*	154 (SG)			2 years	Pain episodes 3 months pre- and post-treatment	Favourable	High	Single-arm assessed.
		(I) UDCA	46.9(1.7)	23:56	10mg/kg/d	59% *versus* 26% (*P* < 0.001)			Satisfactory treatment duration
		(C)* pre- *versus* post-treatment							
Tomida (Japan)^16^	PC	527 (SG + ASG)			66.4(45) months	Reduced risk pain at 10-years	Favourable	Low	+ASG patients.
		(I) UDCA (181 of 527) (74 of 181 SG)	53.4(13.0)	2:3	600mg/d	62%			Satisfactory treatment duration
		(C) No UDCA (346 of 527) (112 of 346 SG)	54.3(12.8)	2:3		92% (*P* < 0.001; RR 0.19; 95% c.i. 0.10,0.34)			
Tint (USA)^17^	PC†	11 (SG + ASG)			6–38 months	Pretreatment colic episodes 22 of 53 (41.5%). 6 months post-treatment 3 of 53 (5.7%) (not reported by dose)	Favourable	Low	+ASG patients
		(I) UDCA	57(13)	6:13	250–300 mg/d				Variable treatment duration
		(C)† pre- *versus* post-treatment	61(10)	11:8	500–600 mg/d				
			55(12)	8:7	900–1000 mg/d				
Polli (Italy)^18^	PC‡	116 (SG + ASG)			6–12 months	Improvement in dyspeptic/pain episodes	Favourable	Low	+ASG patients
									Single-arm assessed.
		(I) UDCA (78 of 116)	51	4:9	5–6/10–12 mg/kg/d	85% pts improvement			Moderate treatment duration
		(C)‡ pre- *versus* post-treatment							
Meredith (UK)^19^	RC§	98 (SG + ASG)			18 months	Colic episodes pre- *versus* during treatment:	Favourable	Low	+ASG patients
		(I) UDCA (46 of 98)	53.2(2.2)	1:2.5	2.5–10 mg/kg/d	72% reduction			Single-arm assessed.
		(C)§ pre- *versus* post- treatment							Satisfactory treatment duration

PC, prospective cohort; RC, retrospective cohort; SG, symptomatic gallstone patients; ASG, asymptomatic gallstone patients; UDCA, ursodeoxycholic acid; CDCA, chenodeoxycholic acid; I, intervention; C, comparator; s.d. standard deviation; mg/d, milligrams per decilitre; mg/kg/d, milligrams per kilogram per decilitre; GRADE, grade of recommendations, assessment, development, and evaluation. *Study comparator CDCA plus UDCA, but not relevant to review. †Study comparator different doses UDCA, but biliary episode occurrence not reported by dose. ‡Study comparator CDCA, but not relevant to review. §Study comparator CDCA, but not relevant to review. GRADE assessment summary designations are as follows: high, very confident that the true effect lies close to that of the estimate of the effect; moderate, moderately confident in the effect estimate (true effect is likely to be close to estimate of effect, but there is a possibility it is substantially different); low, confidence in the effect estimate is limited (true effect may be substantially different from the estimate of the effect); very low, very little confidence in the effect estimate (true effect is likely to be substantially different from the estimate of effect).

## Assessment of bias

### RCTs

**Fig. 4:**
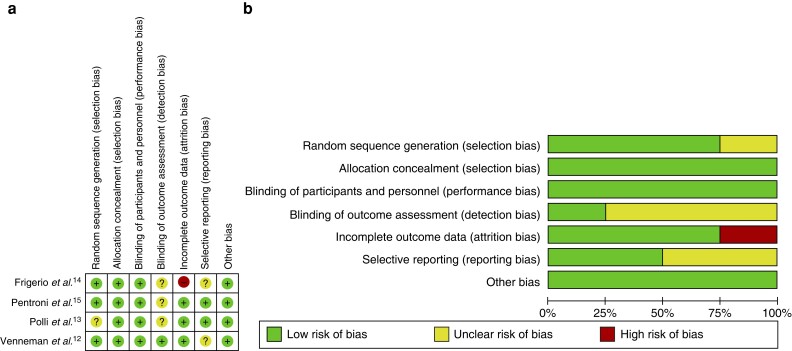
Assessment of risk of bias for randomized clinical studies included in the systematic review **a** Display of individual study performance. **b** Graphical presentation of the compound risk of bias of the three included randomized studies.

The quality of RCTs included in the systematic review is summarized in *Fig. 4*, and all were assessed as having a low risk of bias overall.

### Cohort studies

**Fig. 5:**
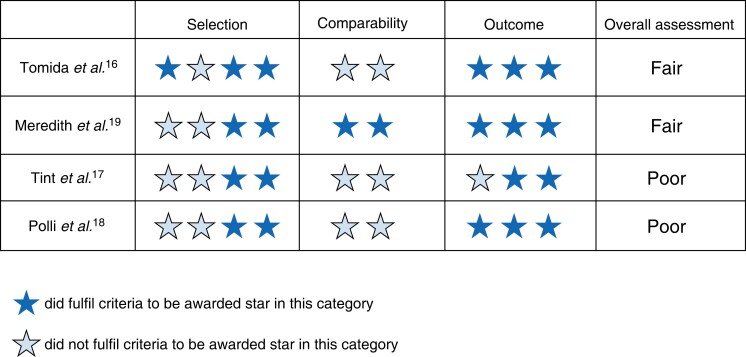
Assessment of cohort studies included in systematic reviews using the Newcastle–Ottawa scale

A summary and breakdown of study quality is shown in *Fig. 5*. None of the four cohort studies achieved a ‘good’ score according to the NOS bias assessment, with two reaching the threshold for ‘fair’, and two ranking as ‘poor’. While the ‘outcome’ criteria are acceptable in the studies, often poor ‘selection’ and ‘comparability’ criteria warrant further investigation of the effect of UDCA in patients with symptomatic gallstone disease.

## Discussion

This systematic review of the use of UDCA for symptomatic gallstones found that there was overall evidence of their beneficial effect. A national clinician survey described the current practice and beliefs regarding UDCA for symptomatic gallstone disease, demonstrating a lack of confidence in such benefits, while identifying clinically meaningful outcomes for future studies.

The present systematic review found that studies varied considerably in design, size, duration of treatment and follow-up, and the demographics of patients, ranging from 42 to 527, some of whom were asymptomatic at recruitment. While a beneficial effect of UDCA in reducing pain episodes and other gallstone-related symptoms was broadly observed in seven of eight studies, results were limited by heterogeneity, variable follow-up and dosing, and subjective endpoints, making interpretation challenging. There was no consistent definition for reporting symptomatic episodes across studies, and variable lengths of follow-up were undertaken, ranging from 14 days to 38 months.

Numerous randomized trials for UDCA and its effect on stone dissolution have been conducted, though only three placebo-controlled studies investigated the impact of UDCA on biliary pain^12–14^. While two studies indicated the benefit of UDCA on reducing gallbladder-related dyspepsia and pain in the short term, their 14-day treatment interval was too short to investigate long-term benefits (or potential adverse effects) of UDCA. Furthermore, the present review identified a risk of bias in these studies, including non-reporting of reasons for dropout (Frigerio *et al*.), minimal discussion on randomization technique, and allocation concealment. The third RCT used a longer treatment interval, with a median follow-up of 77 days before cholecystectomy (range 7–244), and all patients were symptomatic^12^. Overall, 14 per cent (11 of 81) patients in the UDCA group experienced colic episodes within 6 months of commencing therapy, *versus* 7 per cent (6 of 81) in the placebo group. Although having the longest follow-up interval of the available RCTs, and arguably the most rigorous methodology of the papers in this review, follow-up was limited by planned early cholecystectomy at less than 3 months in three-quarters of patients.

All four cohort studies were favourable for UDCA, demonstrating a reduction in incidence of colic episodes or biliary pain^16–19^; however, Tomida *et al.* were not rigorous in their selection process, including only patients who were ‘agreeable’ to commence on UDCA. With those who refused being used as the control group. This exposes this prospective study to substantial risk of selection bias. Equally, Meredith *et al*. selected patients for UDCA therapy ‘carefully’, with ‘ideal’ patients selected ‘deliberately’ (depending on criteria defined by Iser *et al.*^20^). Similarly, a lack of explanation as to their selection process, prohibits Polli *et al.* from a repeatable selection process and exposes the study to risk of bias. While the benefit of the three cohort studies is their longer follow-up, their methodologies demand more robust studies to corroborate their favourable outcomes.

Despite the selection process, Tomida *et al.* reported a significant decrease in complications for patients during 18 years of follow-up and this should not be disregarded. In contrast with Venneman *et al.* who saw no beneficial effects of UDCA *versus* placebo in 6 months, Tomida *et al.* reported a symptom recurrence rate of only 10 per cent in the UDCA cohort, *versus* 40 per cent without. Venneman *et al.* compared their study with Tomida *et al.* and suggested that the different result was attributable to patients in their study having more severe disease. A placebo-controlled randomized study investigating the effect of UDCA on gallbladder contractility and the influence of inflammatory processes culpable in development of biliary pain, may support the hypothesis of such a comparison^21^.

Three studies favourable of UDCA used CDCA rather than placebo as their comparator, therefore the effect on biliary pain was assessed as a single-arm pre- and post-UDCA treatment effect in this review. Moreover, these studies used different doses and regimens of UDCA. Venneman *et al.* prescribed 750 mg/day, whereas other studies used 300 mg/day. Interestingly the study using a higher dose was the only one to report unfavourable results for UDCA. Heterogeneity in dosing strategies and follow-up further highlights the absence of a defined evidence-base regarding the potential benefits of UDCA where greater standardization in future studies is required. Furthermore, future work should explore a dose–response relationship of the effect of UDCA on biliary pain.

Petroni *et al.* reported a significant reduction in biliary pain in both their UDCA alone and combination therapy cohort^15^. Although this RCT was not placebo-controlled and considered as a single-arm study, it is the only study that offered a quantitative change in the incidence of biliary pain, both before and after commencing UDCA in a predefined timeframe, achieving a ‘high’ GRADE score. This reporting structure is one that future studies should emulate, offering a simple and reproducible measure that clinicians can refer to when considering UDCA use.

Variability in definitions of reporting outcomes makes comparing studies challenging. Outcomes across studies include remaining colic-free, number of colic episodes per month, radiation, and number of analgesics taken. To enable clinically relevant comparisons, the use of an internationally accepted measure for biliary colic and severity of symptoms and episodes is necessary. The authors propose the use of an objective primary outcome such as gallstone-related hospital admission would be the most clinically relevant for a future RCT to assess the clinical efficacy of UDCA as a measure of both the impact for the patient and the healthcare system. Using standard definitions for the diagnosis of biliary symptoms such as the Rome criteria for biliary pain and Tokyo guidelines for acute cholecystitis would enable standardization and reduce bias^22,23^.

The results of the clinician survey demonstrate national variation in use of UDCA in patients with symptomatic gallstone disease, with most surveyed clinicians not having used it previously. There was a low awareness of the licenced indication and benefits of UDCA for the management of such patients. More than half of responders were unaware of the published literature on UDCA use in gallstone disease, and half were unsure whether it was of benefit; however, 95 per cent of clinicians reported they would use UDCA if there was an RCT demonstrating benefit, and clinicians felt a reduction in symptomatic episodes and hospital admissions would be the most clinically meaningful outcomes to change practice.

Standard treatment for symptomatic gallstone disease is operative management with a cholecystectomy; however, for those unfit for surgery, UDCA may represent and attractive adjunct to NOM for the frail or surgically unfit. With improvements in access to procedures such as transpapillary endoscopic ultrasound-guided gallbladder drainage, there are increasing minimally invasive options for acute disease that are less morbid than percutaneous drainage; however, these are not a definitive procedures and UDCA may be an ideal adjunct to such procedures to prevent disease recurrence. With global delays to surgery caused by cancellation of operations due to the COVID pandemic, UDCA could represent an important adjunct to operative management where waiting lists are increasing. Venneman *et al.* investigated this and failed to show a clinical benefit, but this is probably due to the high number of cases in the study undergoing cholecystectomy ‘early’ in less than 3 months, before the long-term benefit of UDCA has been realized. Although UDCA has been shown to have a relatively rapid benefit for biliary pain, longer follow-up intervals assessing this effect of UDCA could observe a positive impact on patient quality of life, but such a study is yet to be performed^13,14^.

The most obvious limitation to the review, and thereby the status of evidence of UDCA for biliary pain, is the combination of limited published evidence and heterogeneity of studies. Furthermore, such heterogeneity is in part related to the non-uniformity of definitions of outcome measures. With published definitions and guidelines available now, all studies investigating biliary pain should report outcomes according to those detailed in the Tokyo guidelines^3^.

The clinician survey was disseminated via invitation primarily to surgeons; however, the NOM of gallstone disease is also undertaken by other specialists such as geriatricians and gastroenterologists. Doctors from all specialties involved in gallstone management, particularly for high-risk patients, and more importantly, the patients themselves, should be involved and engaged in any future studies on use, effects, benefits, and acceptability of UDCA. An accurate response rate for the survey is not available given the distribution method of the clinician survey. The denominator of how many clinicians had access to the survey was unattainable due to the use of social media dissemination in addition to e-mail invitation. This could have been mitigated by disseminating the survey through invitation only but this may have limited response numbers and exposed the results to a selection bias. This method may be exposed to response bias, in that those choosing to complete the survey may have had more extreme opinions on UDCA use and retrospective recall of practice may have resulted in a degree of recall bias.

UDCA is licenced for the treatment of gallstones. Clinicians who manage symptomatic gallstone disease are largely unaware of UDCA use in this setting, and many are sceptical of its benefits. The published literature has demonstrated the ability of UDCA to dissolve gallstones, but evidence for its effect in reducing biliary pain is sparse. This review highlights that seven of the eight studies were favourable for the use of UDCA in symptomatic gallstone disease but these studies had considerable flaws. The only study not supportive of UDCA was the most robustly performed (randomized placebo-controlled study of symptomatic patients), though study duration may be insufficient to demonstrate the full effect of UDCA. Almost all survey respondents agreed that an up-to-date RCT is required to improve the evidence. In absence of a non-flawed randomized study, this review concludes that current evidence is in favour of UDCA, but it is not definitive, explaining clinician equipoise. A definitive, randomized trial to investigate the role of UDCA for patients with symptomatic gallstones, particularly in high-risk groups such as the elderly and/or with co-morbid disease unfit for cholecystectomy is urgently needed. The survey data support the primary outcome for such a trial to be the of number of re-admissions to hospital with symptomatic gallstones, and if shown to be beneficial would change practice.

## Supplementary Material

zrac152_Supplementary_Data

## Data Availability

The data that support the findings of this study are available from the corresponding author upon reasonable request.
