# Supplementary material for: Ursodeoxycholic acid in the management of symptomatic gallstone disease: systematic review and clinician survey
Source: BJS Open. 2023 Mar 23;7(2):zrac152. doi: 10.1093/bjsopen/zrac152 (PMC10035564; doi:10.1093/bjsopen/zrac152)
Supplement: zrac152_Supplementary_Data [file zrac152_supplementary_data.docx]

**TItle**

***Current Opinion on Ursodeoxycholic Acid (UDCA) use in the management of Symptomatic Gallstone Disease: Systematic Review of the Literature and Clinician Survey***

Authors

Lewis Hall (1), James Halle-Smith (2), Richard Evans (3), Giles Toogood (4), Tom Wiggins (5), Sheraz Marker (6), Spyros Kapoulas (5), Paul Super (5), Olga Tucker (5) and Siobhan C McKay (2,3)

Affiliations

1. *College of Medical and Dental Scientists, University of Birmingham, United Kingdom*
2. *Liver Unit, Queen Elizabeth Hospital Birmingham, University Hospitals Birmingham United Kingdom*
3. *Department of Academic Surgery, University of Birmingham*
4. *St James’ Hospital, Leeds, United Kingdom*
5. *Department of UGI Surgery, Heartlands Hospital, University Hospitals Birmingham, United Kingdom*
6. *Nuffield Department of Surgery, University of Oxford*

**Corresponding author.** Name and address **ORCID ID**; **Twitter**

Mrs Siobhan McKay

Academic Clinical Lecturer

The Liver Unit, Queen Elizabeth Hospital,

Mindelsohn Way,

Birmingham,

United Kingdom

B15 2TH

Email: [s.mckay@bham.ac.uk](mailto:s.mckay@bham.ac.uk)

ORCID: 0000-0003-0590-2153

Twitter: @siobhanmckay

**Supplementary Materials - Index**

| **Supplementary Methods** |  |
| --- | --- |
| Clinician Survey | *2-7* |
| Search Terms | *8* |
| PRISMA Checklist | *9-11* |
| **References** | *12-13* |
|  |  |

**Supplementary Methods**

**CLINICIAN SURVEY**

Conservative treatment of surgically high-risk patients with symptomatic gallstones: is there a role for ursodeoxycholic acid? Current practice and future perspectives.

Dear Colleague,

For surgically high-risk patients there remain unanswered questions for the optimal conservative management of symptomatic gallstone disease, including ‘would the use of Ursodeoxycholic acid improve outcomes for conservatively managed patients?’

We would be grateful to learn from your practice and experience of using (or not using) Ursodeoxycholic acid for the treatment of symptomatic gallstone disease. This will provide a better understanding of current practice, decision-making and management of symptomatic gallstone disease in surgically high risk patients.

Once the survey is complete we will be holding a meeting to disseminate the results, and invite you to join us to discuss a trial of Urseodeoxycholic acid in the conservative management of surgically high risk patients.

Many thanks,


Siobhan McKay and Professor Giles Toogood
On behalf of the UPLIFT Study Team

* Required

1. Email *

2. First name

3. Surname

4. Current hospital

5. Role

*Mark only one*

Consultant Yr 1-2

Consultant Yr 3-5

Consultant Yr 6-10

Consultant Yr >10

Trainee ST3-8 including OOP

Trainee other

6.What is your defined specialty

*Check all that apply.*

Surgery

General/Acute medicine

Gastroenterology

Care of the elderly

General Practice

Other

If you are a surgeon please answer the questions below and then continue from the page "Conservative management for symptomatic gallstones".

7. Approximately how many cholecystomies are performed in your hospital per year

*Mark only one oval.*

<100

100-200

201-500

501-1000

>1000

8. Please estimate how many patients with symptomatic gallstone disease you personally managed definitively with conservative management annually (prior to COVID)?

*Mark only one oval.*

0-10

11-30

31-50

>50

9. Please estimate what proportion of symptomatic patients you manage don’t receive surgery?

*Mark only one oval.*

1-10%

11-25%

26-50%

51-75%

76-90%

91-100%

If you are a physician please answer the questions in sections and answer all questions thereafter.

10. Please estimate how many patients with symptomatic gallstone disease you managed definitely with conservative management in the year prior to COVID?

*Mark only one oval.*

0-10

11-30

31-50

>50

11. What proportion of patients with symptomatic gallstone disease do you refer for surgical review?

*Mark only one oval.*

0-10%

11-25%

26-50%

51-75%

76-90%

91-100%

CONSERVATIVE MANAGEMENT FOR SYMPTOMATIC GALLSTONES: Please answer all

12. Does the type of gallstone-related disease (e.g. biliary colic, cholecystitis, cholangitis, gallstone pancreatitis) influence whether you offer/refer for elective cholecystectomy vs conservative management to ‘high risk’ patients?

*Mark only one oval.*

Yes

No

13. Do you routinely offer/refer surgically high risk patients who have initially undergone conservative management (acutely) for elective cholecystectomy for the following conditions (please tick as applicable) :

*Check all that apply.*

Biliary colic

Acute cholecystitis

Perforated acute cholecystitis

Gallstone pancreatitis

Cholangitis

ASSESSING PATIENT RISK:

We would like to understand more about the patients factors you base your management decision on to conservatively manage patients.

14. Do you have a specific age that you use as cut-off in your practice above which you would consider cholecystectomy/ referral for cholecystectomy high risk?

*Mark only one oval.*

No

>65 years

>70 years

>80 years

>90 years

15.Do you have a specific performance status that you use as cut-off in your practice above which you would not consider cholecystectomy/referral for cholecystectomy?

*Mark only one oval.*

No

PS 1-Restricted in physically strenuous activity but ambulatory and able to carry out work of a light or sedentary nature, e.g., light house work, office work

PS 2-Ambulatory and capable of all selfcare but unable to carry out any work activities; up and about more than 50% of waking hours

PS 3-Capable of only limited selfcare; confined to bed or chair more than 50% of waking hours

PS 4-Completely disabled; cannot carry on any selfcare; totally confined to bed or chair

16. Do you use any of the following factors in your assessment of a patient symptomatic gallstone disease to determine whether someone is surgically high risk? Tick as appropriate

*Check all that apply.*

No

Age

Performance status

ASA

Frailty

Chronic liver disease

Disseminated cancer

Diabetes mellitus

Steroid use for chronic condition

Hypertension requiring medication

Congestive cardiac failure

Dyspnoea with moderate exertion

Dyspnoea at rest

Current smoker

Severe COPD

Renal failure requiring dialysis

BMI

Fall History

Residential/nursing home resident

Dementia/cognitive impairment

Anticoagulation use

Anaesthetic assessment

17. Risk Assessment Tools: Do you use a surgical risk calculator when assessing risk for the individual patient?

*Check all that apply.*

No

ASA

P-Possum

ACS NSQUIP Surgical risk calculator

Surgical Risk Preoperative Assessment System (SURPAS)

9-point S-MPM (Surgical Mortality Probability Model) 30-day mortality risk index

Revised Cardiac Risk Index

Assessment of METs (Metabolic equivalents)

Cardio-pulmonary testing (C-PEX)

Incremental shuttle walk test (ISWT)

Clinical frailty scale

Edmonton

PRISMA-7

Fried frailty assessment

eFI

18. Have you used UDCA in treatment of symptomatic gallstones?

*Mark only one oval.*

Yes

No

19. If yes, in which of the following cases have you used UDCA?

*Check all that apply.*

Symptomatic disease whilst optimising for surgery (e.g. weight management)

Symptomatic disease for conservatively managed patients - Time limited prescription e.g. 3-6 months, or until gallstone dissolution

Symptomatic disease for conservatively managed patients - Lifelong treatment

20. What dose of UDCA do you use?

*Mark only one oval.*

Standard dose for all patients – please provide total daily dose in mg

Weight-based dosing - please provide the dosing calculation in mg/kg

Other:

21. If other please specify.

22. If standard dose used please specify (Mg)

23. If weight based calculation please specify (Mg/Kg)

24. Dosing regimen

*Mark only one oval.*

OD

BD

TDS

QDS

25. Do you follow-up patients you have started on UDCA to assess efficacy and side-effects?

*Mark only one oval.*

Yes

No

26. If yes, which follow-up technique do you use?

*Check all that apply.*

Clinical assessment

Imaging

27. Do you believe that UDCA can be beneficial in the management of symptomatic gallstones?

*Mark only one oval.*

No

Yes

Unsure

28. Beneficial effects of UDCA in symptomatic gallstone disease

*Check all that apply.*

Reduces the incidence of pain

Reduces the incidence of hospital admissions

Reduce the size of gallstones/dissolves gallstones

Not applicable (I don't believe there are beneficial effects)

29. If you do not use UDCA in symptomatic gallstone disease, why do you not use it?

*Check all that apply.*

Do not believe it works

Unacceptable side effect profile

Too expensive

Concerns about polypharmacy

Not aware of its use in gallstone disease

I manage all of my patients operatively

30. Are you familiar of the literature concerning the use of UDCA and gallstones?

*Mark only one oval.*

Yes

No

31. If you are not using UDCA would you consider it in high risk surgical candidates for conservative management of gallstones?

*Mark only one oval.*

Yes

No

32. If no, why not?

33. What evidence would you need to see to adopt UDCA into routine practice for high risk patients?

*Check all that apply.*

Reduction in episodes of pain

Reduction in requirement for cholecystectomy

Reduction in requirement for percutaneous cholecystostomy

Reduction in hospital admissions

Reduction in incidence of episodes of gallstone related symptoms

Reduction in functional deterioration

Absence of side-effects

Cost effectiveness analysis

Level 1 evidence of benefit

I already use it

34. What is the most important piece of evidence you would need to see to adopt UDCA into routine practice for high risk patients?

*Mark only one oval.*

Reduction in episodes of pain

Reduction in requirement for cholecystectomy

Reduction in requirement for percutaneous cholecystostomy

Reduction in hospital admissions

Reduction in incidence of episodes of gallstone related symptoms

Reduction in functional deterioration

Absence of side-effects

Cost effectiveness analysis?

Level 1 evidence of benefit

I already use it

35. Would you start using UDCA if there was an RCT demonstrating its benefits?

*Mark only one oval.*

Yes

No

36. Would you be interested in participating as an investigator in a RCT testing the efficacy of UCDA in conservative management of gallstones in high risk surgical candidates?

*Mark only one oval.*

Yes

No

37. Would you be willing to randomise your conservatively managed patients to either UDCA or no UDCA for conservative Mx?

*Mark only one oval.*

Yes

No

38. Are you happy to be contacted again to help inform the design of a trial to optimise the conservative management of surgically high risk patients with symptomatic gallstone disease?

*Mark only one oval.*

Yes

No

**SEARCH TERMS**

((((((UDCA[Title/Abstract]) OR (ursodeoxycholic acid[Title/Abstract])) OR (ursodiol[Title/Abstract])) OR (Actigall[Title/Abstract])) OR (Ursofalk[Title/Abstract])) AND (((((gallstone[Title/Abstract]) OR (gall stone[Title/Abstract])) OR (cholelithiasis[Title/Abstract])) OR (biliary calculus[Title/Abstract])) OR (biliary calculi[Title/Abstract]))) AND ((((((((((admission[Title/Abstract]) OR (pain[Title/Abstract])) OR (analgesia use[Title/Abstract])) OR (cholangitis[Title/Abstract])) OR (biliary colic[Title/Abstract])) OR (biliary pain[Title/Abstract])) OR (gallstone pancreatitis[Title/Abstract])) OR (cholecystitis[Title/Abstract])) OR (hospitalization[Title/Abstract])) OR (hospitalisation[Title/Abstract]))

**PRISMA CHECKLIST**

| **Section/topic** | **#** | **Checklist item** | **Reported on page #** |
| --- | --- | --- | --- |
| **TITLE** | | |  |
| Title | 1 | Identify the report as a systematic review, meta-analysis, or both. | 1 of 30 |
| **ABSTRACT** | | |  |
| Structured summary | 2 | Provide a structured summary including, as applicable: background; objectives; data sources; study eligibility criteria, participants, and interventions; study appraisal and synthesis methods; results; limitations; conclusions and implications of key findings; systematic review registration number. | 2 of 30 |
| **INTRODUCTION** | | |  |
| Rationale | 3 | Describe the rationale for the review in the context of what is already known. | 3 of 30 |
| Objectives | 4 | Provide an explicit statement of questions being addressed with reference to participants, interventions, comparisons, outcomes, and study design (PICOS). | 3 of 30 |
| **METHODS** | | |  |
| Protocol and registration | 5 | Indicate if a review protocol exists, if and where it can be accessed (e.g., Web address), and, if available, provide registration information including registration number. | N/A |
| Eligibility criteria | 6 | Specify study characteristics (e.g., PICOS, length of follow-up) and report characteristics (e.g., years considered, language, publication status) used as criteria for eligibility, giving rationale. | 4 of 30 |
| Information sources | 7 | Describe all information sources (e.g., databases with dates of coverage, contact with study authors to identify additional studies) in the search and date last searched. | 4 of 30 |
| Search | 8 | Present full electronic search strategy for at least one database, including any limits used, such that it could be repeated. | Sup. File 2 |
| Study selection | 9 | State the process for selecting studies (i.e., screening, eligibility, included in systematic review, and, if applicable, included in the meta-analysis). | 4 of 30 |
| Data collection process | 10 | Describe method of data extraction from reports (e.g., piloted forms, independently, in duplicate) and any processes for obtaining and confirming data from investigators. | 4 of 30 |
| Data items | 11 | List and define all variables for which data were sought (e.g., PICOS, funding sources) and any assumptions and simplifications made. | 5 of 30 |
| Risk of bias in individual studies | 12 | Describe methods used for assessing risk of bias of individual studies (including specification of whether this was done at the study or outcome level), and how this information is to be used in any data synthesis. | 5 of 30 |
| Summary measures | 13 | State the principal summary measures (e.g., risk ratio, difference in means). | N/A |
| Synthesis of results | 14 | Describe the methods of handling data and combining results of studies, if done, including measures of consistency (e.g., I^2^) for each meta-analysis. | N/A |

| **Section/topic** | **#** | **Checklist item** | **Reported on page #** |
| --- | --- | --- | --- |
| Risk of bias across studies | 15 | Specify any assessment of risk of bias that may affect the cumulative evidence (e.g., publication bias, selective reporting within studies). | 5 of 30 |
| Additional analyses | 16 | Describe methods of additional analyses (e.g., sensitivity or subgroup analyses, meta-regression), if done, indicating which were pre-specified. | N/A |
| **RESULTS** | | |  |
| Study selection | 17 | Give numbers of studies screened, assessed for eligibility, and included in the review, with reasons for exclusions at each stage, ideally with a flow diagram. | 6 & 22 of 30 |
| Study characteristics | 18 | For each study, present characteristics for which data were extracted (e.g., study size, PICOS, follow-up period) and provide the citations. | 7 & 8 of 30 |
| Risk of bias within studies | 19 | Present data on risk of bias of each study and, if available, any outcome level assessment (see item 12). | 8 of 30 |
| Results of individual studies | 20 | For all outcomes considered (benefits or harms), present, for each study: (a) simple summary data for each intervention group (b) effect estimates and confidence intervals, ideally with a forest plot. | 16 & 17 of 30 |
| Synthesis of results | 21 | Present results of each meta-analysis done, including confidence intervals and measures of consistency. | N/A |
| Risk of bias across studies | 22 | Present results of any assessment of risk of bias across studies (see Item 15). | 23 & 24 of 30 |
| Additional analysis | 23 | Give results of additional analyses, if done (e.g., sensitivity or subgroup analyses, meta-regression [see Item 16]). | N/A |
| **DISCUSSION** | | |  |
| Summary of evidence | 24 | Summarize the main findings including the strength of evidence for each main outcome; consider their relevance to key groups (e.g., healthcare providers, users, and policy makers). | 9-11 of 30 |
| Limitations | 25 | Discuss limitations at study and outcome level (e.g., risk of bias), and at review-level (e.g., incomplete retrieval of identified research, reporting bias). | 12 of 30 |
| Conclusions | 26 | Provide a general interpretation of the results in the context of other evidence, and implications for future research. | 12 of 30 |
| **FUNDING** | | |  |
| Funding | 27 | Describe sources of funding for the systematic review and other support (e.g., supply of data); role of funders for the systematic review. | 1 of 30 |

*From:*  Moher D, Liberati A, Tetzlaff J, Altman DG, The PRISMA Group (2009). Preferred Reporting Items for Systematic Reviews and Meta-Analyses: The PRISMA Statement. PLoS Med 6(7): e1000097. doi:10.1371/journal.pmed1000097

**References**

1. Aerts R, Penninckx F. The burden of gallstone disease in Europe. Aliment Pharmacol Ther. 2003;18 Suppl 3:49-53.

2. Guarino MP, Cocca S, Altomare A, Emerenziani S, Cicala M. Ursodeoxycholic acid therapy in gallbladder disease, a story not yet completed. World J Gastroenterol. 2013;19(31):5029-34.

3. Yokoe M, Hata J, Takada T, Strasberg SM, Asbun HJ, Wakabayashi G, et al. Tokyo Guidelines 2018: diagnostic criteria and severity grading of acute cholecystitis (with videos). J Hepatobiliary Pancreat Sci. 2018;25(1):41-54.

4. NICE. Gallstone disease: diagnosis and management: Clinical guideline [CG188] 2014 [Available from: <https://www.nice.org.uk/guidance/cg188>.

5. Wiggins T, Markar SR, Mackenzie H, Jamel S, Askari A, Faiz O, et al. Evolution in the management of acute cholecystitis in the elderly: population-based cohort study. Surg Endosc. 2018;32(10):4078-86.

6. Kamarajah SK, Karri S, Bundred JR, Evans RPT, Lin A, Kew T, et al. Perioperative outcomes after laparoscopic cholecystectomy in elderly patients: a systematic review and meta-analysis. Surg Endosc. 2020;34(11):4727-40.

7. Surgeons call for investment in surgical hubs as NHS waiting list hits record 5.3 million [press release]. <https://www.rcseng.ac.uk/news-and-events/media-centre/press-releases/waiting-times-may-2021/>, 08/07/2021 2021.

8. Portincasa P, Di Ciaula A, Wang HH, Moschetta A, Wang DQ. Medicinal treatments of cholesterol gallstones: old, current and new perspectives. Curr Med Chem. 2009;16(12):1531-42.

9. Nasser M. Cochrane Handbook for Systematic Reviews of Interventions. Am J Public Health. 2020;110(6):753-4.

10. Guyatt GH, Oxman AD, Vist GE, Kunz R, Falck-Ytter Y, Alonso-Coello P, et al. GRADE: an emerging consensus on rating quality of evidence and strength of recommendations. BMJ. 2008;336(7650):924-6.

11. Wells G SB, O’Connell D, Peterson J, Welch V, Losos M, Tugwell P. The Newcastle-Ottawa Scale (NOS) for assessing the quality of nonrandomised studies in meta-analyses 2013 [Available from: <http://www.ohri.ca/programs/clinical_epidemiology/oxford.asp>.

12. G F. Ursodeoxycholic Acid (UDCA) in the Treatment of Dyspepsia: Report of a

Multicenter Controlled Trial. . Curr Ther Res. 1979(26):214-23.

13. Polli E I-H. Ursodeoxycholic Acid (UDCA) in the Treatment of Dyspepsia: Report of a

Multicenter Controlled Trial. . Curr Ther Res 1979(26):230-4.

14. Venneman NG, Besselink MG, Keulemans YC, Vanberge-Henegouwen GP, Boermeester MA, Broeders IA, et al. Ursodeoxycholic acid exerts no beneficial effect in patients with symptomatic gallstones awaiting cholecystectomy. Hepatology. 2006;43(6):1276-83.

15. Polli EE, Bianchi PA, Conte D, Sironi L. Treatment of radiolucent gallstones with CDCA or UDCA. A multicenter trial. Digestion. 1981;22(4):185-91.

16. Tomida S, Abei M, Yamaguchi T, Matsuzaki Y, Shoda J, Tanaka N, et al. Long-term ursodeoxycholic acid therapy is associated with reduced risk of biliary pain and acute cholecystitis in patients with gallbladder stones: a cohort analysis. Hepatology. 1999;30(1):6-13.

17. Meredith TJ, Williams GV, Maton PN, Murphy GM, Saxton HM, Dowling RH. Retrospective comparison of 'Cheno' and 'Urso' in the medical treatment of gallstones. Gut. 1982;23(5):382-9.

18. Tint GS, Salen G, Colalillo A, Graber D, Verga D, Speck J, et al. Ursodeoxycholic acid: a safe and effective agent for dissolving cholesterol gallstones. Ann Intern Med. 1982;97(3):351-6.

19. Iser JH, Dowling H, Mok HY, Bell GD. Chenodeoxycholic acid treatment of gallstones. A follow-up report and analysis of factors influencing response to therapy. N Engl J Med. 1975;293(8):378-83.

20. Guarino MP, Cong P, Cicala M, Alloni R, Carotti S, Behar J. Ursodeoxycholic acid improves muscle contractility and inflammation in symptomatic gallbladders with cholesterol gallstones. Gut. 2007;56(6):815-20.

21. Petroni ML, Jazrawi RP, Pazzi P, Lanzini A, Zuin M, Pigozzi MG, et al. Ursodeoxycholic acid alone or with chenodeoxycholic acid for dissolution of cholesterol gallstones: a randomized multicentre trial. The British-Italian Gallstone Study group. Aliment Pharmacol Ther. 2001;15(1):123-8.

22. Drossman DA. Functional Gastrointestinal Disorders: History, Pathophysiology, Clinical Features and Rome IV. Gastroenterology. 2016.

23. Mayumi T, Okamoto K, Takada T, Strasberg SM, Solomkin JS, Schlossberg D, et al. Tokyo Guidelines 2018: management bundles for acute cholangitis and cholecystitis. J Hepatobiliary Pancreat Sci. 2018;25(1):96-100.
